# Network analyses reveal the role of large snakes in connecting feeding guilds in a species‐rich Amazonian snake community

**DOI:** 10.1002/ece3.7508

**Published:** 2021-05-01

**Authors:** Daniela Pinto‐Coelho, Marcio Martins, Paulo Roberto Guimarães Junior

**Affiliations:** ^1^ Departamento de Ecologia Instituto de Biociências Universidade de São Paulo São Paulo Brazil

**Keywords:** body size, Boidae, complex networks, *Eunectes murinus*, modularity, nestedness, trophic interaction

## Abstract

In ecological communities, interactions between consumers and resources lead to the emergence of ecological networks and a fundamental problem to solve is to understand which factors shape network structure. Empirical and theoretical studies on ecological networks suggest predator body size is a key factor structuring patterns of interaction. Because larger predators consume a wider resource range, including the prey consumed by smaller predators, we hypothesized that variation in body size favors the rise of nestedness. In contrast, if resource consumption requires specific adaptations, predators are expected to consume distinct sets of resources, thus favoring modularity. We investigate these predictions by characterizing the trophic network of a species‐rich Amazonian snake community (62 species). Our results revealed an intricate network pattern resulting from larger species feeding on higher diversity of prey and therefore promoting nestedness, whereas snakes with specific lifestyles and feeding on distinct resources, promoting modularity. Species removal simulations indicated that the nested structure is favored mainly by the presence of five species of the family Boidae, which because of their body size and generalist lifestyles connect modules in the network. Our study highlights the particular ways traits affect the structure of interactions among consumers and resources at the community level.

## Graphical abstract

Network describing the interactions (lines) between species of Amazonian snakes (circles) and their food resources (triangle). The colors represent food modules that group species that consume similar sets of resources.

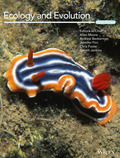

## 1 INTRODUCTION

Interactions between species are a key component for understanding biodiversity (Abram, 1987). In fact, individuals of all species rely upon ecological interactions to obtain food, to breed, or to protect against natural enemies (Thompson, 2005). Ecological interactions form networks that connect populations of different species in a locality (Bascompte & Jordano, 2013). The organization of these networks may have important conservation implications, potentially affecting the robustness of ecological systems to species loss (Schmitz & Beckerman, 2007). In this context, it is essential to understand how factors that influence the interactions between individuals affect the structure of networks at the level of ecological communities.

The structure of several ecological networks generally deviates from what is expected for networks in which individuals interact randomly; that is, the interaction is proportional to the product of species abundances (Krishna et al., 2008). These deviations from expected network structure suggest that factors such as the characteristics of interacting individuals and environmental conditions influence the structural patterns of ecological networks at the community level. Among the traits that may affect network structure is body size, which is directly associated with the ability of individuals to consume resources (Stouffer et al., 2011). At the species level, there is strong evidence that diet width, that is, the number of different resources that organisms of a given species feed on, varies with species mean body size, as observed in mammals (Sinclair et al., 2003), frogs (Ceron et al., 2019), fish (Rezende et al., 2009), and birds (Gliwicz, 2008). If on average the larger the predator, the greater its ability to kill larger prey, we should expect that, at the species level, larger average body sizes to be correlated with a larger number of resource types consumed by predator species (i.e., large predator species will have a diet that includes both small and large prey) (Sinclair et al., 2003). Furthermore, if only body size were influencing the capacity to consume a wider range of resources, it is expected that the diet of the smaller predator species would be a subset of the items of the larger predator species' diet, leading to nested ecological networks (Sinclair et al., 2003; Stouffer et al., 2011; Woodward et al., 2005).

On the other hand, food resources are not homogeneously distributed in environments and the degree of specialization in the consumption of distinct sets of prey may require distinct adaptations (Covich & McDowell, 1996; Schoener, 1968). For instance, the *Anolis* lizards of South Bimini islands divide habitat and the food resources according to lizard average size classes, in which larger lizard species usually eat larger food items than smaller lizard species (see Schoener, 1968). Thus, we can expect that due to the restrictions related to prey handling, prey detection, or nutritional yield, larger predators are predisposed to disregard smaller prey (Arim et al., 2010; Arnold, 1993; Mittelbach, 1981). In this sense, deviations from the perfectly nested pattern are expected, enabling the formation of semi‐isolated groups (modules) in the network. Networks with a modular structure have stimulated much interest due to its possible evolutionary and ecological consequences (Ings et al., 2009). For instance, modules may represent coevolutionary units (Thompson, 2005) and increase the stability of ecological networks, thus providing a potential mechanism through which complexity arises and persists in ecological communities (Ings et al., 2009; Krause et al., 2003).

Here, we explore the trophic network organization of a community of Amazonian snakes. Many snakes are top‐level carnivores that perform important roles in ecosystems (Yanosky et al., 1996). Indeed, snakes have been used as a model system in studies on the effect of ecological interactions on diversity (Alencar et al., 2013, 2017; Bellini et al., 2015; Colston et al., 2010; Klaczko et al., 2016; Martins et al., 2001). These studies explore how ecological traits, interspecific interactions, habitat use, and evolutionary history influence the current trophic interactions of different species. Furthermore, as snakes evolved morphological and behavioral adaptations to kill and ingest their prey whole (Greene, 1983), traits related to different dietary habits of species make snakes a model study system to understand how trophic interactions organize community structure (Shine & Bonnet, 2000). In addition, some species (e. g., those in the genera *Bothrops*, *Crotalus*, *Lachesis*, and *Micrurus*) are of medical importance (Campbell & Lamar, 1989) and knowledge about their trophic ecology may favor studies focusing on public health; for example, diet patterns are related to poison chemistry (see Daltry et al., 1996; Davies & Arbuckle, 2019).

Motivated by understanding the trophic organization pattern of snake communities, we here use as a model a rich and well‐studied community of Amazonian snakes (Martins & Oliveira, 1998). We characterized the structure of the interaction network between snakes and their food resources. We expected that if only snake body size were shaping network patterns at the species level, the structure would be nested; on the other hand, if specialization in resource consumption were driving patterns of resource use across snakes, modularity would be expected. We then evaluated the role of different snake species and the effect of snakes' habitat use (referred to here as a lifestyle) in shaping the network structure.

## 2 METHODS

### 2.1 The network structure of interactions between snakes and their food resources

We analyzed the snake diet derived from a long‐term study carried out in a Central Amazonia site on the natural history of forest snakes (Martins & Oliveira, 1998). We described the resource use by snakes as an interaction matrix **A** in which if a snake *i* feeds on a given resource *j* and zero otherwise. The matrix **A** defines a bipartite network in which one set of nodes is represented by snake species and the other set of nodes by resource types and the links describe interactions between snake species and food resource types. Our food resources are not described at the species level, but at coarse categories such as small mammals, medium mammals, and big mammals (see details in Appendix S2 dataset). Similar approach led to insights into the study of food webs (Cohen, 1977) and individual‐based networks (Araújo et al., 2008). In fact, there is no intrinsically correct level of description when characterizing an ecological network (Guimarães, 2020). We opted for these coarse categories because they are in agreement with (a) the evidence that snakes are specialized in broad categories of resources, for example, serpentiform organisms that include snakes, amphisbaenians, and caecilians (see Martins & Oliveira, 1998); and (b) the level of detail available from the diet analyses of snakes. Having said that, to verify whether our level of network description affects our analyses we performed a set of sensitivity analyses (details below).

We used four metrics to characterize the structure of the interactions network analyzed: (a) degree distribution, which is the description on how the number of food resources a given snake can feed on (the degree) varies across snake species; (b) connectance (*C*), which is the proportion of all possible interactions actually recorded in the network. Connectance values range from 0 (nonconnected network) to 1 (maximum connectance); (c) modularity (*M*), a measure of the extent to which the network is formed by groups (modules) of snake species in which snake within a module overlap in much of their resources, whereas snakes in different modules show no or weak resource use overlap; and (d) nestedness (N), which consists of an interaction pattern in which the specialists interact with sets of resources with which the generalists also interact. Detailed descriptions of the metrics are available in the Appendix S1.

We used *Q_B_* metric, defined by Barber (2007), to characterize modularity, with values ranging from 0 (nonmodular network) to 1 (completely modular). A simulated annealing algorithm (Guimerà & Amaral, 2005) was used to optimize the *Q_B_* value. Modularity analyses were performed using the Modular program (Marquitti et al., 2014). All the above and the following analyses were performed using R version 3.5.1 (R Core Team, 2020), with the exception of modularity. We performed a set of sensitivity analyses to verify whether our results are dependent on our approach to compute modularity (Appendix S1).

The NODF metric was used to characterize the nestedness degree (Almeida‐Neto et al., 2008), and its values range from 0 (non‐nested network) to 100 (perfect nestedness). The degree of nestedness and modularity was then compared with a theoretical benchmark provided by the null model 2 of Bascompte et al. (2003) (see detailed description in Appendix S1). We generated 1,000 null model matrices to estimate nestedness and modularity. If a network shows a degree of nestedness or modularity larger than expected by the null model 2, then there is evidence of ecological or evolutionary processes acting on the network organization that goes beyond those shaping the degree of specialization of the snake species (e.g., Bascompte et al., 2003).

In order to highlight the unique inferences provided by the network approach, we compared the results of the network analysis with the results of a multivariate analysis. Multivariate analysis methods are widely used in ecology due to their ability to analyze complex systems registered in an interaction matrix (Prado et al., 2002). Among the several types of multivariate analyses, we chose correspondence analysis (CA) because of its ability to reveal reciprocal relationships between two sets of equal interest (Greenacre, 1984; Lewinsohn et al., 2006), in our case, snakes and their food resources.

### 2.2 The role of snake species in network structure

If the network of interactions analyzed follows the organization pattern structured by body mass (i.e., presenting higher nestedness than expected by the null model 2), we hypothesized snake average body mass to be positively correlated with the number of resources consumed by the snake species. To explore this prediction, we investigate the association between average body mass and the role of species in the network structure. We recorded the estimates of the average body mass of each snake species in our network (data available in Feldman et al., 2016). Average body mass was log‐transformed prior to analysis (Appendix S2: Table A1).

In order to understand the individual contribution of each species of snake to nestedness, we used a jackknife resampling approach in which we removed a snake species and recomputing the degree of nestedness. We repeated the procedure for all snake species in the network and then we computed a change in nestedness: Δ*N_i_ =* *N* − *N_i_,* in which *N* is the degree of nestedness of the complete network and *N_i_* is the degree of nestedness after the removal of a snake species *i*. If body size were shaping the contribution to nestedness, we should expect that the Δ*N_i_* would assume increasingly positive values as larger snakes are removed from the network, indicating that nestedness is higher in the presence of these larger snake species.

### 2.3 The relationship between lifestyles and network structure

Because dietary specialization in snakes can be related to habitat occupation (see Alencar et al., 2017; Martins et al., 2002), we expect snake lifestyles to affect the degree of dietary specialization (e.g., an aquatic snake would rely upon aquatic prey). If this is true, the distribution of lifestyles in the different modules will not be random. We evaluated this prediction using two analyses. First, we analyzed the frequency of snake lifestyles in different modules. We estimated the probability of the observed number of species of a given lifestyle in a given module be reproduced by randomly assigning species across modules, but preserving the number of snake species in each lifestyle and the number of snake species in each module (*n* = 1,000 randomizations). Then, we analyzed the dissimilarity on lifestyles between modules. To do so, we used the Bray–Curtis index, available in the vegan package in R (Oksanen et al., 2020) (see detailed description in Appendix S1). Dissimilarity between a pair of modules ranges from 0 (modules are identical in the composition of lifestyles) to 1 (no lifestyle occurs in both modules).

### 2.4 Sensitivity analyses focus on the level of resource resolution

Sampling effects may affect the description of network patterns. Therefore, we performed a sensitivity analysis to explore how robust is the description of network patterns to changes in our dataset. We add information to the use of resources by snakes by using data from other Amazonian regions, based on evidence that there is no significant intraspecific variation on the snake's diet across different localities in Amazonia (Martins & Oliveira, 1998; Appendix S1 and Appendix S2: Table A1).

Snake diet often includes food resources that are mainly consumed and resources that are only eventually consumed. We performed a sensitivity analysis to check whether the patterns reported in our study are robust enough when considering the presence or absence of secondary resources in the snake diet. We described two matrices of interactions: (a) a matrix in which only main resources were considered; and (b) and a matrix in which both main and secondary resources were considered. We defined whether a resource is main or secondary according to information about snake diet preferences available in Martins and Oliveira (1998). Then, we calculate the nestedness and modularity values in the presence and absence of secondary resources. The nestedness values of the two networks were compared with a null model generated with 5,000 random removals of food resources from each of the analyzed networks. Finally, we calculated whether there was a significant difference between the nestedness of the network in the presence and absence of secondary resources.

Because taxonomic resolution might influence the detection of patterns in the network (Rezende et al., 2009), we performed another sensitivity analysis to check whether the type of resource categorization could affect the network patterns. Thus, we described two other matrices of interaction with different degrees in the resources of taxonomic resolution: less specific network (Appendix S1: Figure A2 and Appendix S3: Table A1) and more specific network (Appendix S1: Figure A3 and Appendix S4: Table A1).

## 3 RESULTS

### 3.1 Network structure

**FIGURE 1:**
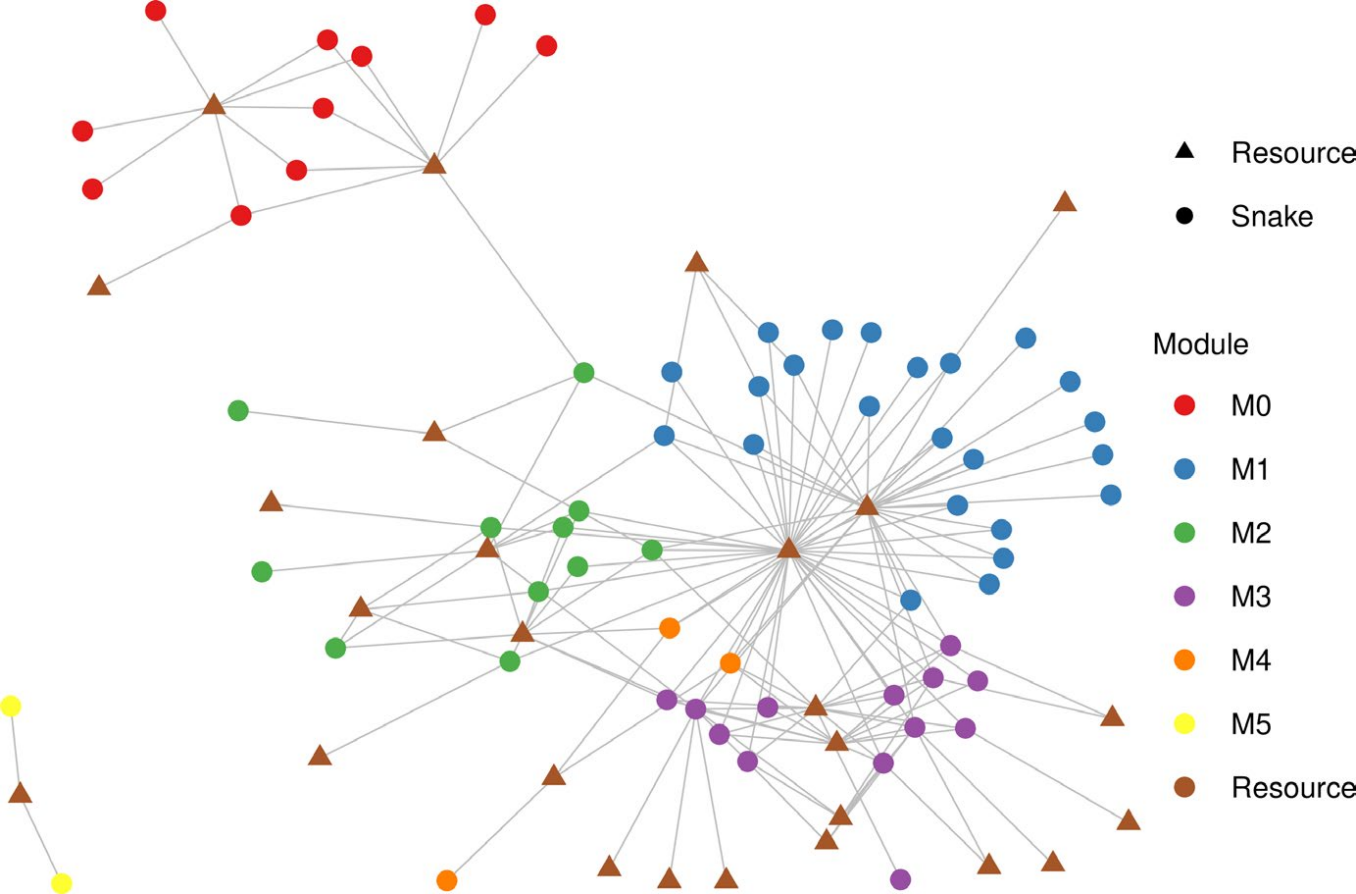
Network describing the interactions (lines) between species of Amazonian snakes (circles) and their food resources (triangle)

We recorded 163 interactions between 62 snake species and 26 food resources (Figure 1 and Appendix S1: Figure A6 ) that were heterogeneously distributed among snake species, where most of them had few interactions (56.45% snake species interacted with one or two resource categories) and few species had many interactions (6.45% interacted with more than five resources; Appendix S1: Figure A1). The network structure shows moderate connectance (C = 0.101) (Table 1), indicating that, from the variety of food items consumed by snakes, the species analyzed use, on average, 2–3 resources. The snake‐resource network also shows significant nestedness (*N* = 33.14, *p* < .01), indicating that 1/3 of the interactions of the less connected species represent a subset of the interactions of the most connected species. Finally, the network also shows significant modularity (*M* = 0.51, *p* = .03), indicating that the number of interactions within each module is 51% larger than what is expected for a network with the same number of modules, the same number of interactions per species, but with random interactions between species.

**TABLE 1:** Relationship of the network structure analysis of interactions between Amazonian snakes and their food resources

Web	S	R	SD	C	N	*p* N	Nrel	M	*p* M	nM	Mrel
Complete web	62	26	2.62	0.10	33.14	*p* < .01	0.94	0.51	*p* = .03	6	0.11
Without secondary resources	62	24	2.34	0.09	29.46	*p* < .01	0.93	0.48	*p* = .44	7	0.03
Without the 7 largest species	55	18	2.23	0.12	30.23	*p* < .01	0.69	0.53	*p* = .06	7	0.12
Without species of Boidae	57	19	2.26	0.12	30.42	*p* < .01	0.74	0.53	*p* = .01	7	0.13

Abbreviations: C, connectivity; M, modularity; Mrel, relative modularity; N, nestedness; nM, number of food modules. Nrel, relative nestedness; R, food resources richness (note that with the removal of species from the network occurs the loss of interactions, which may reduce the number of resources); S, snake species richness in the network; SD, average degree.

Some snake species showed extreme specialization, such as *Dipsas* spp., which feed exclusively on mollusks, and *Drepanoides anomalus* that rely upon eggs of squamate reptiles. Other species, such as *Atractus* spp., although specialist in the consumption of earthworms, may also feed on insects. Similarly, *Micrurus surinamensis* primarily consume fish but secondarily consume lizards and snakes. On the other hand, we found very generalist species, such as *Boa constrictor* and *Epicrates cenchria*, which interacted with six resource types, *Corallus hortulanus*, which interacted with eight resources and *Eunectes murinus*, the largest species of the network, which interacted with 11 resources. Among the food resources consumed by many snake species were lizards (24% of all interactions), anurans (16%), and small mammals (9%), comprising rodents and marsupials. Among the least consumed resources were large mammals (such as cervids), turtles and alligators, only consumed by *Eunectes murinus*, onychophorans only consumed by *Micrurus hemprichii*, Gymnophiona only consumed by *M. lemniscatus*, and salamanders, which were only consumed by *Chironius fuscus* (Appendix S2: Table A1).

To assess whether there was a difference in network structure based only on the presence of primary resources in the snake diet, we removed all nonprimary resources and reanalyzed the network. Even after removing the secondary resources, network average degree and connectance remained within the same range values (Table 1). The results also indicated that there was no significant difference between the network nestedness values with and without the presence of secondary resources (*p* = .147). Even after the removal of nonprimary items, the network remained significantly nested (*N* = 29.46, *p* < .01). In contrast, the modular structure was nonsignificant after removal of nonprimary resources (*M* = 0.47, *p* = .44). Similarly, to check whether the type of food resource categorization could affect the network patterns, we used the same metrics to analyze the more specific and the less specific networks. Our results for all, but connectance, hold with different levels of detail on resource description, and all networks remained significantly nested and modular. Connectance was the only metric that values show large variation across levels of detail on resource description, and connectance increased (Appendix S1: Table A2).

**FIGURE 2:**
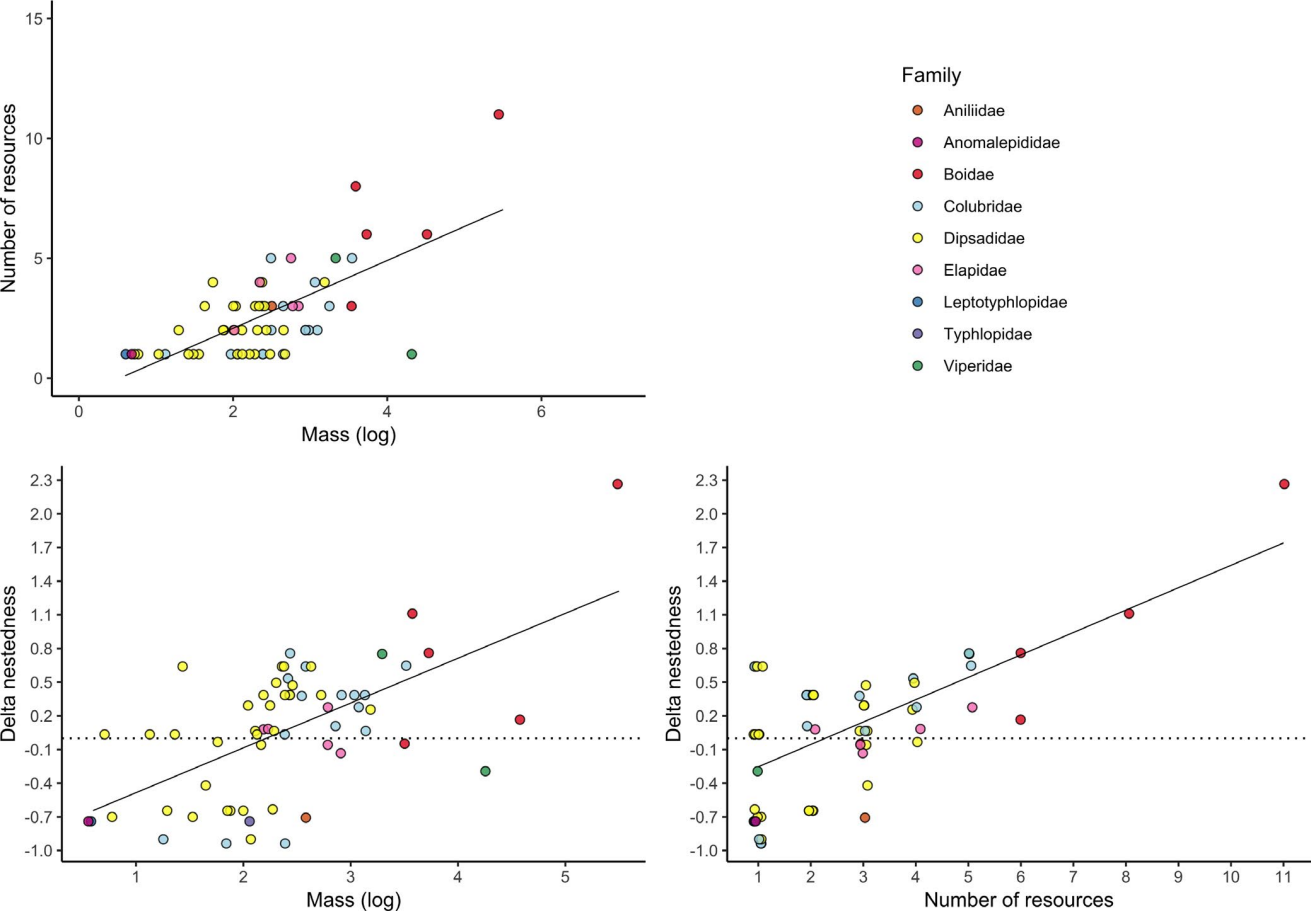
(a) The number of categories of food resources consumed by different snake species is positively associated with the snake average body mass (slope = 1.41, *R* ^2^ = 0.46, *p* < .01) in a network of interactions between Amazonian snakes and their resources. In simulations of species removal, (b) changes in the level of nestedness are positively associated with the snake average body mass of the removed species (slope = 0.40, *R* ^2^ = 0.37, *p* < .01), (c) and with the number of food resources (degree) (slope = 0.19, *R* ^2^ = 0.46, *p* < .01). Note the stronger, negative changes in nestedness are associated with Boidae snakes (red). Each point represents a species, and the colors represent the different snake families. To avoid overlap, some points have been slightly offset from their original position on the *x*‐axis

Our results supported the prediction that there is a positive association between the number of resources consumed and average body size (slope = 1.41, *R* ^2^ = 0.46, *p* < .01, Figure 2a), indicating that in general the largest species of snakes showed a greater number of food interactions. Exceptions to this pattern were *Corallus caninus* (k = 3) and *Lachesis muta* (k = 1), both specialists in the consumption of mammals. Among the seven largest snake species, five of them (*Eunectes murinus*, *Boa constrictor*, *Epicrates cenchria*, *Corallus hortulanus*, and *Corallus caninus*) belong to the family Boidae. Thus, this family is overrepresented among the set of heavier snakes in the network and our analysis may be biased by the confounding factors generated by all other traits shared by boid species. To circumvent this problem, we explored whether the correlation between average body mass and degree holds within speciose snake families. We performed correlation analyses between degree and average body mass for species of the family Colubridae and for those of the family Dipsadidae, the two largest snake families in the network. The results indicated that a positive correlation between average body mass and the number of resources consumed hold even for non‐boid snakes and partially controlling for phylogenetic effects (see Appendix S1: Figure A4 and Figure A5).

### 3.2 The role of different species of snakes in network structure

The correlation between degree and average body mass suggests nestedness was driven by body size. In fact, there is a positive correlation between delta nestedness and average body mass (slope = 0.4, *R* ^2^ = 0.37, *p* < .01, Figure 2b), as well as between delta nestedness and degree (slope = 0.19, *R* ^2^ = 0.46, *p* < .01, Figure 2c), indicating that the largest snakes have a greater contribution to nestedness. We performed a removal analysis to further explore this pattern. We removed species with more outlier values of average body mass and recalculated the nestedness value. They were the seven largest snake species in the network (*Eunectes murinus*, *Boa constrictor*, *Lachesis muta*, *Epicrates cenchria*, *Corallus hortulanus*, *Corallus caninus*, and *Spilotes pullatus*). If the largest snakes are key components contributing to nestedness, we expected that nestedness after removal of these species would be smaller than those generated by a null model in which we randomly remove any seven species from the network and recalculate the nestedness. Our results supported this prediction, indicating that the nestedness values were smaller after the removal of the seven largest snake species (*N* = 30.23, *p* < .01, *n* = 1,000 simulations of species removal).

### 3.3 The relationship between modularity and snake lifestyle

**FIGURE 3:**
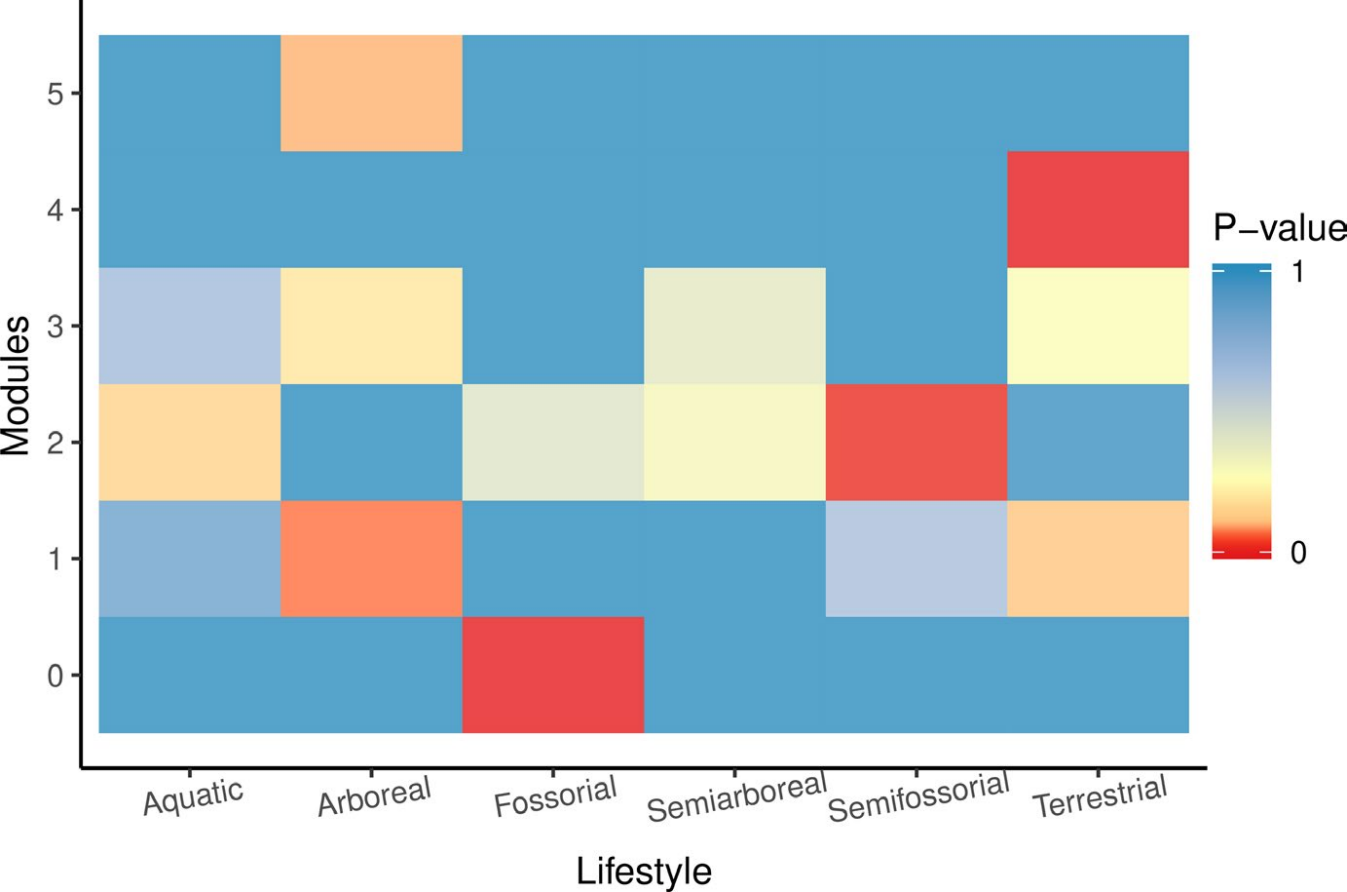
The probability of the observed number of species of a given lifestyle in a given module of being reproduced randomly. Red color indicates higher frequency, with a 95% confidence interval

The network also presented a modular structure, in which the consumption of different resources divided the network into six different food modules (Table 1 and Appendix S1: Table A3). The formation of groups was also observed in our correspondence analysis, although a small number of groups were detected (Appendix S1). We expected that if the snake lifestyle was related to the formation of food modules, the distribution of lifestyles in the different modules would not be random. In fact, modules were associated with particular lifestyles, as indicated by significant or marginally significant probability values present in all modules but module 3 (Table 2). Module 3 was composed of species with the greatest variety in diet and lifestyle, such as boid snakes. Modules with more specific combinations of lifestyle and diet showed all significant or marginally significant probability values (Figure 3). Moreover, the formation of groupings based on diet and lifestyle occurred by species that specialize in the consumption of certain food resources, probably associated with their lifestyle. For example, the smallest module (number 5) was formed by only two arboreal species of the genus *Dipsas* that feed exclusively on mollusks; another module grouped species of terrestrial habits, such as *Drepanoides anomalus*, *Drymoluber dichrous*, and *Mastigodryas boddaerti*, which feed on squamate eggs, whereas another module grouped fossorial species, such as *Atractus* spp. that are specialists in preying upon earthworms. On the other hand, the remaining modules grouped species with varied lifestyles and generalist diets.

**TABLE 2:** Comparison between the real interaction matrix, the dissimilarity matrix, and the likelihood matrix of the lifestyle by food module of the network of interactions between Amazonian snakes and their food resources

Real matrix							
Modules	Aquatic	Arboreal	Fossorial	Semi‐arboreal	Semi‐fossorial	Terrestrial	*n* species
0	0	0	10	0	0	0	10
1	1	9	0	0	2	11	23
2	2	0	3	1	3	2	11
3	1	5	0	1	0	6	13
4	0	0	0	0	0	3	3
5	0	2	0	0	0	0	2
*n* species	4	16	13	2	5	22	62

Dissimilarity matrix						
Modules	0	1	2	3	4	5
0	0	1	0.714	1	1	1
1	1	0	0.706	0.333	0.769	0.840
2	0.714	0.706	0	0.667	0.714	1
3	1	0.333	0.667	0	0.625	0.733
4	1	0.769	0.714	0.625	0	1
5	1	0.840	1	0.733	1	0

Probability matrix						
Modules	Aquatic	Arboreal	Fossorial	Semi‐arboreal	Semi‐fossorial	Terrestrial
0	1	1	0*	1	1	1
1	0.839	0.06*	1	1	0.622	0.121
2	0.152	1	0.41	0.303	0.035*	0.969
3	0.647	0.197	1	0.386	1	0.276
4	1	1	1	1	1	0.027*
5	1	0.078*	1	1	1	1

Note

Lines represent the six (0–5) food modules, and columns represent the lifestyles of the snakes. Asterisks represent significant or marginally significant values of probability.

As a consequence of the association between lifestyles and modular structure, modules often show dissimilar combinations of lifestyles. Dissimilarity values ranged from 0.33 to 1 (Table 2). The most dissimilar module (module 0) was composed exclusively of snakes with the fossorial lifestyle, such as the *Atractus* species, specialists in the consumption of earthworms. The most similar modules were modules 1 and 3, which as well as module 2, group a largest variety of lifestyles and food resources consumed by the snake species contained in these modules (Appendix S1: Table A3).

## 4 DISCUSSION

Our results indicated that the network of interactions between snakes and their resources in a species‐rich Amazonian community presented a combination of both nested and modular structures. Nestedness was related to average body mass of snakes, in which boid snakes connect food modules in the trophic network. The modular pattern, in turn, is associated with the different snake lifestyles, in which snakes that share similar habits usually consume similar resources available in their shared microhabitats.

The observed connectance of the network indicated that, given the variety of resources available in the environment, snake species consumed only a subset of these resources. This result suggests that most food resources may not be accessible to most species, suggesting forbidden interactions (Olesen et al., 2010) associated with possible restrictions related to lifestyle (see Savitzky, 1983), as well as body size (Sinclair et al., 2003; Stouffer et al., 2011; Woodward et al., 2005). For instance, arboreal snakes have morphological adaptations, such as a slender body and long tail, which may represent limitations to the consumption of large prey such as mammals (Alencar et al., 2013, 2017; Martins et al., 2001). The analysis of network structure revealed that the patterns of resource use by different species lead, at the community level, to nestedness and modularity. Our results contrast with some studies on antagonistic networks that indicate opposite trends between nestedness and modularity (Pires & Guimarães, 2012; Thébault & Fontaine, 2010). Having said that, other studies simultaneously show levels of nestedness and modularity (Bellay et al., 2011; Flores et al., 2013; Pinheiro et al., 2019). The emergence of these combined network patterns is possible due to the low connectivity of the network (Fortuna et al., 2010; Lewinsohn et al., 2006) and resource heterogeneity (Pinheiro et al., 2019) in Amazonian forests.

Several processes may explain the nested patterns, such as variations in species abundances (Lewinsohn et al., 2006). One of the explanations for the nested pattern found in our study was the large size variation among species present in the network. The variation in snake body mass has led to a trophic hierarchy in which larger predators prey upon more resource items than smaller predators. This hierarchy was detected in several predator–prey interaction networks found in nature (Arim et al., 2010; Smith & Mills, 2008; Woodward et al., 2005). This pattern indicates that predators have the potential to add resources sequentially as they increase in size, although this increment of larger resources may lead to the rejection of smaller, less nutritious, or difficult to handle resources (Arim et al., 2010; Arnold, 1993; Mittelbach, 1981; Woodward et al., 2010). In addition to body size, skull morphology is also an important feature associated with diet and snake lifestyle (Klaczko et al., 2016; Pough & Groves, 1983; Savitzky, 1983). The larger the head of a snake, the greater the prey consumed (King, 2002). Thus, future research that investigates emerging patterns arising from the association of both body size and skull morphology with the structure of trophic interaction networks could predict the processes, at the community level, involved in the relationships between snakes and their food resources.

When analyzing the contribution of each species to nestedness, we found that average body mass has a phylogenetic signal, with large species concentrated in a few clades. After removing larger species, mostly boids, the nestedness value decreases 8.78% although it still remains significant. The maintenance of nestedness after the removal of large snakes might be a consequence of the number of resource–body mass association holds for smaller snake species, such as colubrid and dipsadid snakes. Boids are efficient constrictors with generalist diets, which occupy diverse microhabitats, which allow them to consume a wide variety of food resources (Henderson & Pauers, 2012; Pizzato et al., 2009). This combination of features may simultaneously explain why (a) boids act as hubs (species with many interactions) in the analyzed network, and (b) the decrease in nestedness when boids are removed from the network. Large predators, such as sharks, killer whales, lions, and birds of prey, often prey on diverse array of species (Sinclair et al., 2003), potentially connecting modules in networks (e.g., Rezende et al., 2009). The fact that a trophic network is connected implies that, at least from a theoretical point of view, indirect effects can propagate across species (Guimarães et al., 2017). As a consequence, the entire assemblage is more sensitive to change (see Andreazzi et al., 2018). In this sense, the highly connected species that create these links among guilds are candidates to play a key role to the ecological and evolutionary dynamics. This potential role is illustrated by our removal simulations that show the potential consequences of the removal of highly connected species to the structure of the network. Future research could test whether the presence of such large predators can also promote nestedness on predator–prey interaction networks.

The modular structure in ecological networks may be associated with factors such as the degree of specialization among interacting species (Lewinsohn et al., 2006; Prado & Lewinsohn, 2004), habitat heterogeneity (Pimm & Lawton, 1980), the phylogenetic relationship between species (Lewinsohn et al., 2006), the convergence in a set of species traits (Olesen et al., 2007), or by a combination of factors (Donatti et al., 2011). We found that the consumption of specific resources is associated with more peculiar lifestyles. For instance, morphological adaptations to fossorial habit (e.g., less cranial mobility) hinder the consumption of prey larger than the snake's head size (Greene, 1983; Martins & Oliveira, 1993; Savitzky, 1983). Accordingly, arboreal habits impose physical limitations on snake morphology and may restrict the consumption of larger prey, such as small mammals, favoring a diet based on lizards and/or frogs (Alencar et al., 2013; Lillywhite & Henderson, 1993; Martins et al., ,^2001^, ^2002^). Thus, we suggest that the modularity of the network we studied has emerged from the relationship between the lifestyles of snakes and the consumption of resources restricted to the habitats used by the species.

To sum up, we integrate network structure analyses with species removal simulations to evaluate the role of different snake species in the structure of a rich Amazonian snake community, and the mechanisms underlying the patterns found. The use of the network approach to understanding the organization of ecological systems provides two sets of insights. First, nonrandom network patterns may represent the fingerprints of ecological and evolutionary processes shaping ecological systems (Guimarães, 2020). In this sense, our quantitative predictions in terms of network descriptors allowed us to reveal how body size, past evolutionary history, and the lifestyles of snakes organize this species‐rich snake assemblage. In this context, modularity is an example of a network descriptor that has been shown to better describe patterns of group organization than other, more traditional multivariate approaches (Amaral & Guimerà, 2005). Accordingly, network plots allow us to have a broader and faster visualization of patterns that would be difficult to observe without using this approach (Marai et al., 2019). Figure 1, for example, allows us to quickly observe that there are two food items consumed by most species of snakes and that there are guilds that are completely specialized in the use of specific resources and others that are connected to the rest of the network by connector species. Second, network description allows us to infer about the robustness of ecological systems and their potential implications for biodiversity conservation (Schmitz & Beckerman, 2007). For example, the presence of connector species allows us to infer about the possibility of the propagation of indirect effects in the network (Guimarães et al., 2017), which could affect species that do not directly interact with each other.

We encourage future studies to focus on understanding how community phylogenetic diversity may be associated with the modular structure (Rezende et al., 2009), as well as how the combination of traits associated with predator diet (e.g., its correlation with body size and skull shape) may contribute to the nested pattern and whether geographic variation (environment type) can modify network structure (Kortsch et al., 2019; Pimm & Lawton, 1980). This study points to the joint importance of the evolutionary history of lineages, body size, and their interacting resources to determine the structure, at the community scale, of the interactions between consumers and their resources.

## CONFLICT OF INTEREST

None declared.

## AUTHOR CONTRIBUTIONS

**Daniela Pinto Coelho:** Conceptualization (lead); Data curation (lead); Formal analysis (lead); Investigation (lead); Methodology (lead); Writing‐original draft (lead). **Marcio Martins:** Data curation (supporting); Funding acquisition (lead); Supervision (supporting); Visualization (supporting); Writing‐original draft (supporting); Writing‐review & editing (supporting). **Paulo R. Guimarães Jr.:** Conceptualization (lead); Data curation (supporting); Formal analysis (supporting); Funding acquisition (lead); Investigation (supporting); Methodology (lead); Project administration (lead); Resources (lead); Supervision (lead); Visualization (lead); Writing‐original draft (lead); Writing‐review & editing (lead).

## Supporting information

Supplementary Material

Supplementary Material

Supplementary Material

Supplementary Material

Supplementary Material

Supplementary Material

Supplementary Material

Supplementary Material

Supplementary Material

Supplementary Material

Supplementary Material

## Data Availability

We declare that all data supporting the conclusions of this study (dataset and supplementary information) are uploaded in Dryad (https://doi.org/10.5061/dryad.f1vhhmgvt).
