# Supplementary figures and images for: Network analyses reveal the role of large snakes in connecting feeding guilds in a species‐rich Amazonian snake community
Source: Ecol Evol. 2021 May 1;11(11):6558–68. doi: 10.1002/ece3.7508 (PMC8207408; doi:10.1002/ece3.7508)

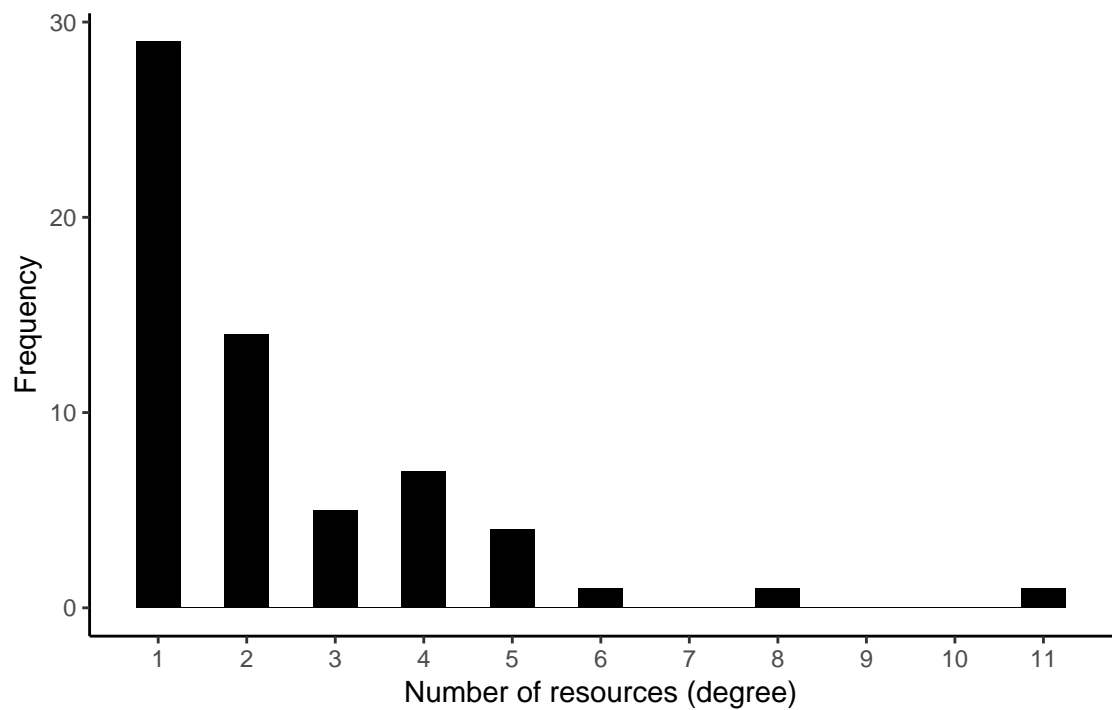

Supplement: Supplementary file 1 — Supplementary Material [file ECE3-11-6558-s010.pdf]

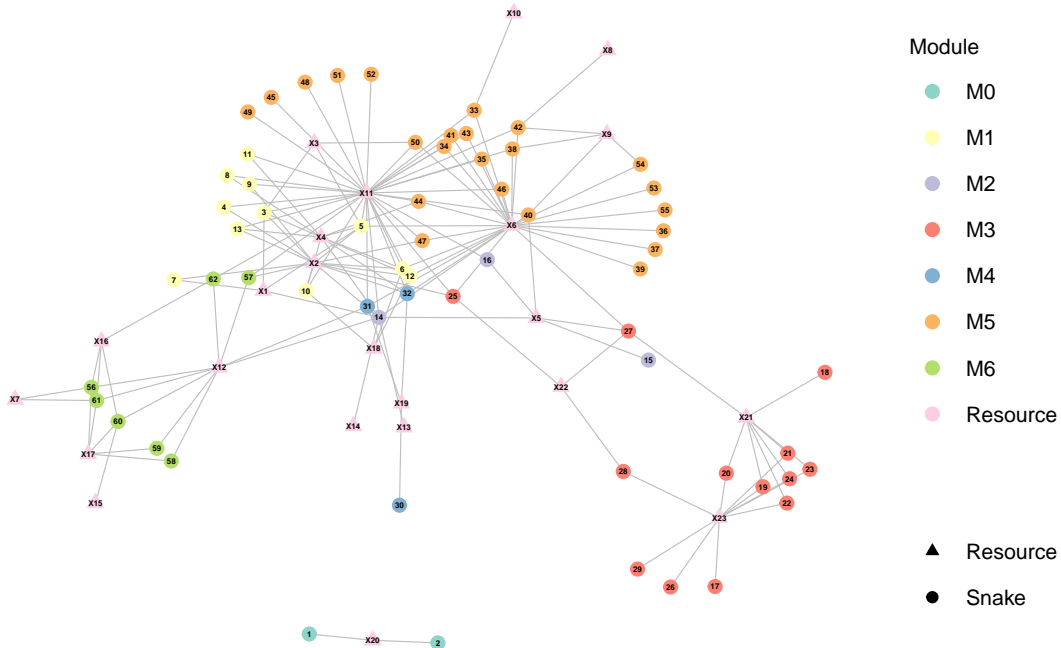

Supplement: Supplementary file 2 — Supplementary Material [file ECE3-11-6558-s003.pdf]

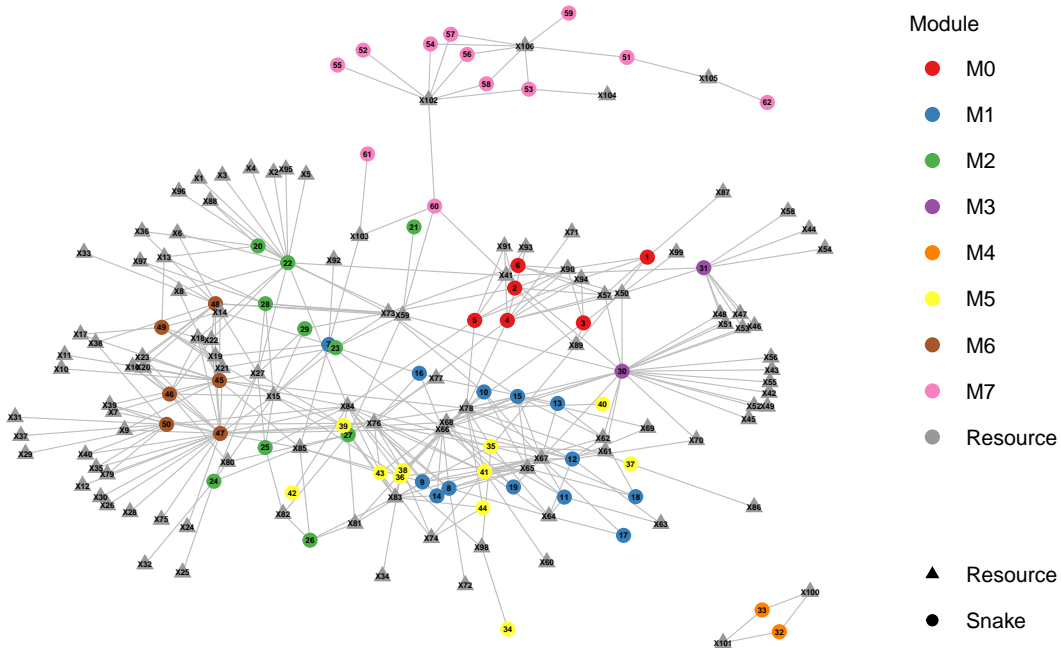

Supplement: Supplementary file 3 — Supplementary Material [file ECE3-11-6558-s009.pdf]

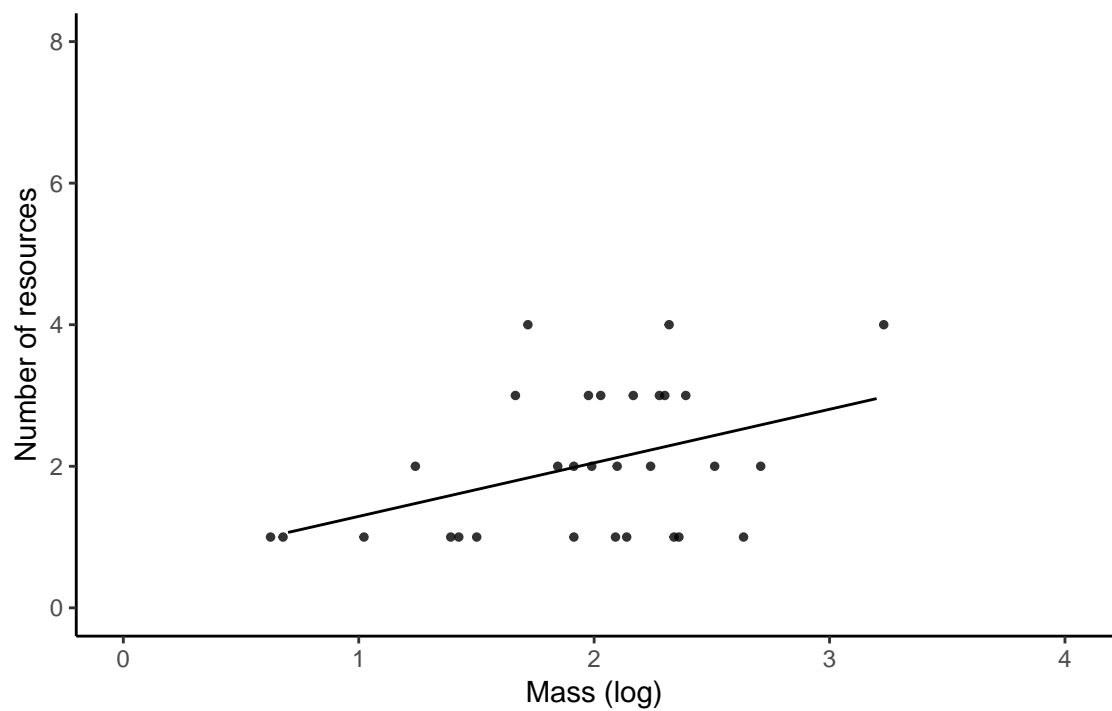

Supplement: Supplementary file 4 — Supplementary Material [file ECE3-11-6558-s001.pdf]

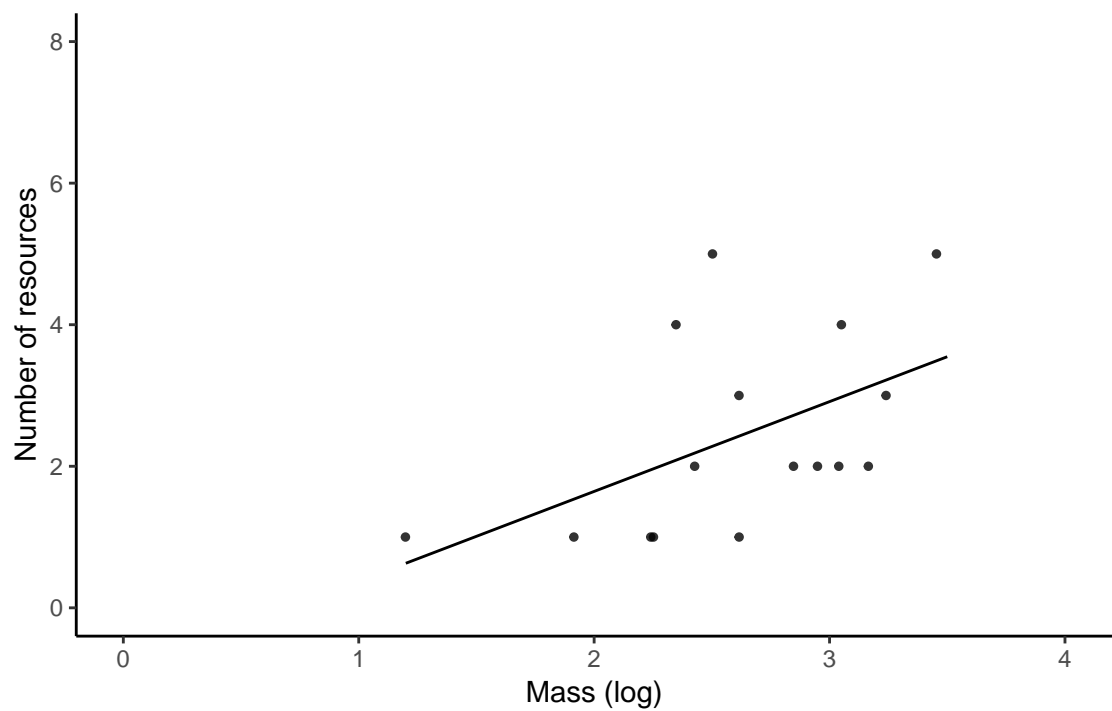

Supplement: Supplementary file 5 — Supplementary Material [file ECE3-11-6558-s005.pdf]

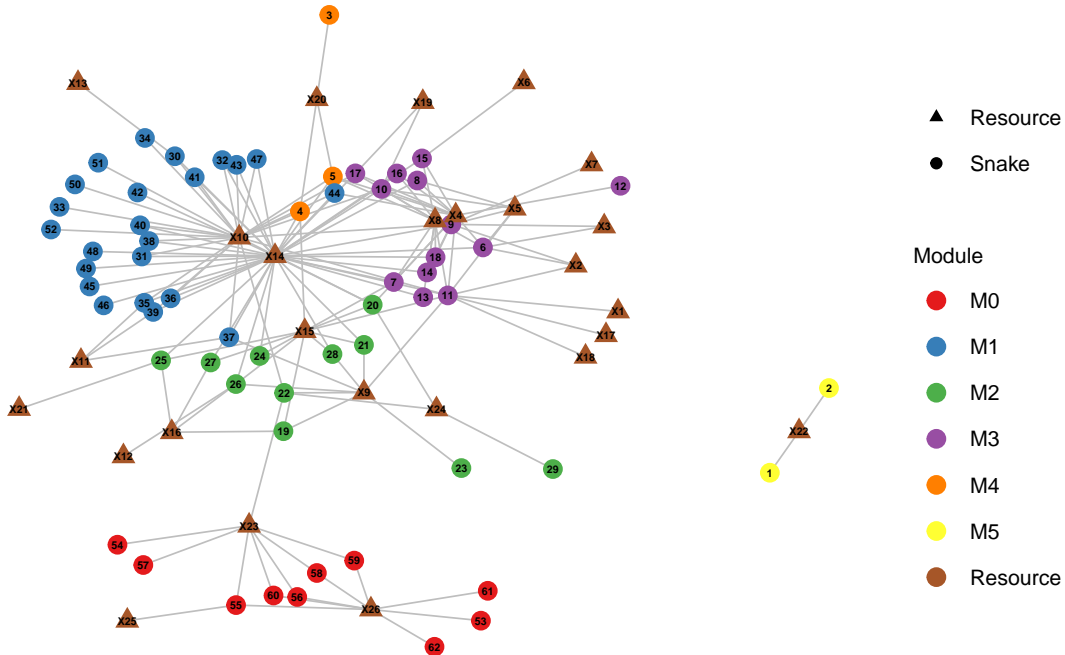

Supplement: Supplementary file 6 — Supplementary Material [file ECE3-11-6558-s002.pdf]

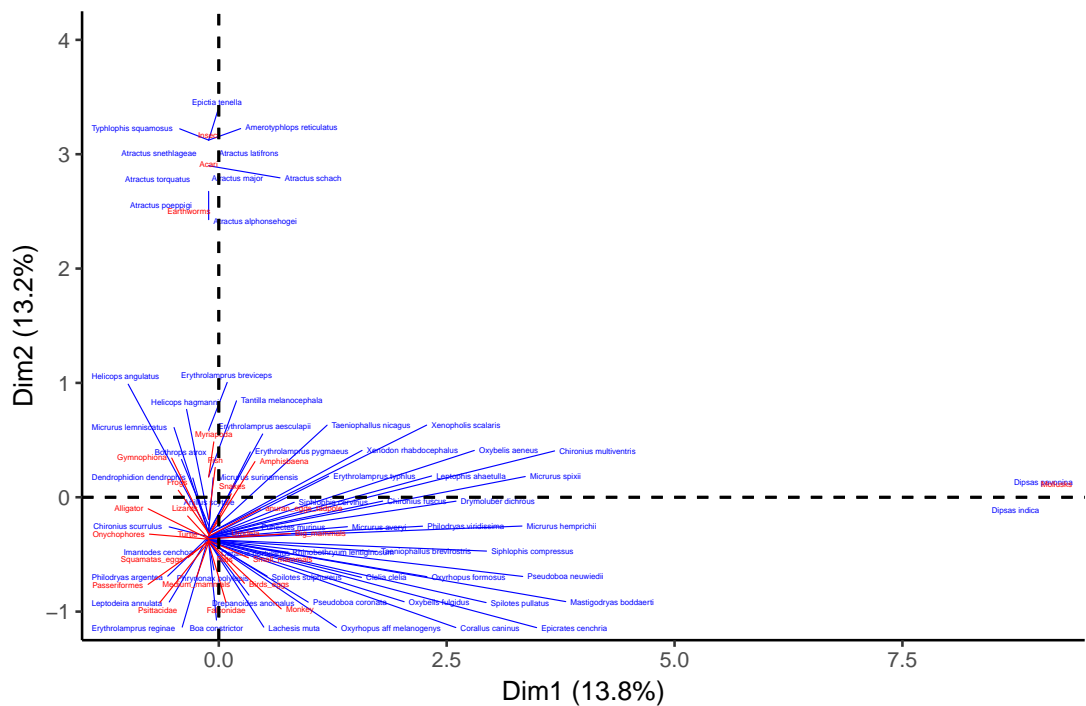

Supplement: Supplementary file 7 — Supplementary Material [file ECE3-11-6558-s011.pdf]
