# Supplementary material for: Network analyses reveal the role of large snakes in connecting feeding guilds in a species‐rich Amazonian snake community
Source: Ecol Evol. 2021 May 1;11(11):6558–68. doi: 10.1002/ece3.7508 (PMC8207408; doi:10.1002/ece3.7508)
Supplement: Supplementary file 8 — Supplementary Material [file ECE3-11-6558-s008.docx]

**Appendix 1**

A detailed description of methods and network metrics used in our study.

1. ***Degree distribution***

The degree of a snake species is the total number of food resources that a snake consumes. The degree distribution is a description of how the number of food resources that a particular snake feeds on varies across snake species.

1. ***Connectance***

The connectance is the proportion of all possible interactions actually recorded in the network:

$C=\frac{I}{SR}$ *,*

where *I* is the number of interactions recorded among snake species and their food resources; *S* is the number of snake species in the network; and *R* is the number of food resources in the network. Connectance values range from 0 (non-connected network) to 1 (maximum connectance).

1. ***Modularity***

The modularity is a measure of the extent to which the network is formed by groups (modules) of snake species in which snake within a module overlap in much of their resources, whereas snakes in different modules show no or weak resource use overlap. We used *Q_B_* metric, defined by Barber (2007), to characterise modularity, with values ranging from 0 (non-modular network) to 1 (completely modular):

$QB=\frac{1}{2I}\sum_{i,j}^{S+R} \left( A_{ij}-P_{ij} \right)\delta\left( g_{i},g_{j} \right)$,

where, *I* is the number of interactions; *A_ij_* is a square and symmetric matrix describing the interactions among snakes and resources, in which $a_{ij}=a_{ji}=1$ if a snake species *i* feeds on a resource *j* and zero otherwise; *P_ij_* is the probabilities in the null model that an interaction exists between vertices *i* and *j*; and $\delta\left( g_{i},g_{j} \right)=1$ if nodes (snakes or resources) *i* and *j* are assigned to the same module and zero otherwise. In the null model, the probability there is an interaction between a snake species and a resource is proportional to the products of the degree of both the resource and the snake species. A simulated annealing algorithm (Guimerà & Amaral 2005) was used to optimise the *Q_B_* value. Modularity analyses were performed using the Modular program (Marquitti et al. 2014). Because our approach to computing modularity is based on optimization of *Q_B_*-values, we performed a sensitivity analysis to check the accuracy of the estimated *Q_B_*, where we calculated 30 times the modularity value for the snakes-prey network and calculated the mean and standard deviation of these results. Moreover, estimates of modularity are often approach-dependent. To verify if our results are dependent on our approach to compute modularity, we compared the performance of the simulated annealing algorithm with the DIRTLPAwb+ algorithm proposed by Beckett (2016) (Table A1).

Table A1: Comparison between simulated anneling, DIRTLPAwb+, and LPAwb+ algorithms in snakes-prey interaction networks.

| **Metric** | **Algorithm** | **Author** | **Modularity** |
| --- | --- | --- | --- |
| Barber (2007) | simulated annealing | Guimerà & Amaral (2005) | 0.50661 |
| Barber (2007) | DIRT LPA wb+ | Beckett (2016) | 0.50562 |
| Barber (2007) | LPA wb+ | Beckett (2016) | 0.45835 |

1. ***Nestedness***

The nestedness is an interaction pattern in which the specialists interact with sets of resources with which the generalists also interact. The NODF (nestedness metric based on overlap and decreasing fill) metric was used to characterise the nestedness degree (Almeida-Neto et al. 2008) and its values ranges from 0 (non-nested network) to 100 (perfect nestedness):

NODF = $\frac{\sum_{i,j}^{S} N_{ij}+\sum_{d,f}^{R} N_{df}}{S\left( S-1 \right)+R\left( R-1 \right)}$ ,

Where $N_{ij}$ is the pairwise degree of nestedness (Almeida-Neto et al. 2008) calculated for snake species i and j and $N_{df}$ is the pairwise degree of nestedness between resources *d* and *f*.

**(e) *Null model***

We compared the degree of nestedness and modularity with a theoretical benchmark provided by the null model 2 of Bascompte et al. (2003). In this null model 2, the probability of a species of snake *i* interact with a resource *j* is

$Pij=\frac{1}{2}\left( \frac{ki}{R}+\frac{kj}{S} \right)$ ,

in which k*_i_* (k*_j_*) is the degree of the snake species *i* (resource *j*), *R* is the number of resource types in the network, and *S* is the snake species richness. We chose the null model 2 because the probability of interaction is proportional to the number of interactions (degree), therefore allowing to investigate if the levels of modularity and nestedness are higher than expected by the variation in number of resources types across snake species.

**(f) Correspondence Analysis (CA)**

Several multivariate methods can be used to elucidate the structure of complex interactions. Among them, CA is adequate to reveal reciprocal relationships between two sets of equal interest (see Lewinsohn and Prado 2008), in our case, snakes and their food resources. We used CA to assess the ability of a multivariate approach to detect the trophic interactions structure in the analysed snakes community.

The results showed that the CA did not clearly identify the existence of the nested pattern and identified only the formation of three food modules (as opposed to the six found by the network analysis). The first module is made of slug-eating snakes of the genus *Dipsas*, the second is formed by earthworm, mite and insect-eating fossoreal snakes, and the third is composed of the all other species, which includes both specialist and generalist snakes.

Unlike the network approach, the graphical visualisation of a multivariate analysis is not so intuitive, without clearly demonstrating the patterns of interaction (Figure A7). For example, it is not possible to observe the number of interactions per species of snake, or which food resources are most consumed, or which interactions connect subgroups, nor to infer about the individual role of species in the community. Therefore, the interpretation of the results from the interaction matrix becomes less straightforward. Thus, we conclude that network analysis is more efficient than multivariate analysis in identifying both nested and modular patterns, in addition to being a visual tool that provides an easier interpretation of interactions.

**(g) *Bray-Curtis index***

To evaluate the hypothesis that the distribution of lifestyles in the different modules will not be random, we analysed the dissimilarity between modules using the Bray-Curtis index, available in vegan package in R (Oksanen et al. 2018). In the Bray-Curtis index,

$I_{ij}=1-\left( \frac{2Cij}{Si+Sj} \right)$ ,

where C*_ij_* is the number of lifestyles in common between both modules, and S*_i_* and S*_j_* are the total number of lifestyles counted at both modules. Dissimilarity values range from 0 (smallest dissimilarity among modules) to 1 (largest dissimilarity among modules).

**Refereces**

Almeida-Neto, M. et al. 2008. A consistent metric for nestedness analysis in ecological systems: reconciling concept and measurement. – Oikos 117: 1227–1239.

Barber, M. J. 2007. Modularity and community detection in bipartite networks. – Phys. Rev. E 76: 066102.

Bascompte, J. et al. 2003. The nested assembly of plant-animal mutualistic networks. – PNAS 100: 9383–9387.

Beckett, S. J. 2016. Improved community detection in weighted bipartite networks. – R. Soc. Open Sci. 3: 140536.

Guimerà, R. and Amaral, L. A. 2005. Functional cartography of complex metabolic networks. – Nature 433: 895–900.

Lewinsohn, T. M. and Prado, P. I. 2006. Structure in plant-animal interaction assemblages. – Oikos 113: 174–184.

Marquitti, F. M. D. et al. 2014. MODULAR: Software for the autonomous computation of modularity in large network sets. – Ecography 37: 221–224.

Oksanen, J. et al. 2018. vegan: Community Ecology Package. R package version 2.5-2. https://CRAN.R-project.org/package=vegan.

Table A2: Comparison between three different degrees of refinement of the networks structure analysis of the interaction between Amazonian snakes and their food resources. S = snake species richness in the network; R = food resources richness (Note that with the removal of species from the network occurs the loss of interactions, which may reduce the number of resources); SD = average degree; C = connectivity; N = nestedness; Nrel = relative nesting; M = modularity; Mrel = relative modularity; nM = number of food modules.

| **Less refined network** | **S** | **R** | **SD** | **C** | **N** | **p N** | **Nrel** | **M** | **p M** | **nM** | **Mrel** |
| --- | --- | --- | --- | --- | --- | --- | --- | --- | --- | --- | --- |
| Complete web | 62 | 23 | 2.50 | 0.11 | 28.64 | p < 0.01 | 0.73 | 0.52 | p < 0.01 | 7.00 | 0.14 |
| Without secondary resources | 62 | 21 | 1.90 | 0.09 | 19.68 | p < 0.01 | 0.63 | 0.60 | p = 0.01 | 9.00 | 0.15 |
| Without the 7 largest species | 55 | 20 | 2.20 | 0.11 | 25.18 | p < 0.01 | 0.61 | 0.56 | p = 0.01 | 7.00 | 0.14 |
| Without species of Boidae | 57 | 21 | 2.20 | 0.11 | 25.21 | p < 0.01 | 0.66 | 0.55 | p = 0.03 | 6.00 | 0.13 |
| **Network with intermediate refinement** | **S** | **R** | **SD** | **C** | **N** | **p N** | **Nrel** | **M** | **p M** | **nM** | **Mrel** |
| Complete web | 62 | 26 | 2.62 | 0.10 | 33.14 | p < 0.01 | 0.94 | 0.51 | p = 0.03 | 6.00 | 0.11 |
| Without secondary resources | 62 | 24 | 2.34 | 0.09 | 29.46 | p < 0.01 | 0.93 | 0.48 | p = 0.44 | 7.00 | 0.03 |
| Without the 7 largest species | 55 | 18 | 2.23 | 0.12 | 30.23 | p < 0.01 | 0.69 | 0.53 | p = 0.06 | 7.00 | 0.12 |
| Without species of Boidae | 57 | 19 | 2.26 | 0.12 | 30.42 | p < 0.01 | 0.74 | 0.53 | p = 0.01 | 7.00 | 0.13 |
| **More refined network** | **S** | **R** | **SD** | **C** | **N** | **p N** | **Nrel** | **M** | **p M** | **nM** | **Mrel** |
| Complete web | 62 | 106 | 5.69 | 0.05 | 13.43 | p < 0.01 | 0.66 | 0.59 | p < 0.01 | 8.00 | 0.25 |
| Without secondary resources | 62 | 104 | 5.35 | 0.05 | 12.98 | p < 0.01 | 0.65 | 0.59 | p < 0.01 | 7.00 | 0.22 |
| Without the 7 largest species | 55 | 79 | 4.72 | 0.06 | 12.71 | p < 0.01 | 0.47 | 0.60 | p < 0.01 | 9.00 | 0.23 |
| Without species of Boidae | 57 | 82 | 4.79 | 0.06 | 12.23 | p < 0.01 | 0.45 | 0.60 | p < 0.01 | 7.00 | 0.21 |

Table A3: Grouping of snake species into food modules, the resources that grouped the modules and lifestyles. F = fossorial; T = terrestrial; Arb = Arboreal; Aq = Aquatic; SF = semifossorial; and SA = semiarboreal.

| **Species** | **Module** | **Resources** | **Lifestyles** |
| --- | --- | --- | --- |
| *Amerotyphlops reticulatus* | 0 | Acari, Earthworm, and Insect | F |
| *Atractus alphonsehogei* | 0 |  | F |
| *Atractus latifrons* | 0 |  | F |
| *Atractus major* | 0 |  | F |
| *Atractus poeppigi* | 0 |  | F |
| *Atractus schach* | 0 |  | F |
| *Atractus snethlageae* | 0 |  | F |
| *Atractus torquatus* | 0 |  | F |
| *Epictia tenella* | 0 |  | F |
| *Typhlophis squamosus* | 0 |  | F |
| *Chironius fuscus* | 1 | Anuran, Anuran eggs and tadpole, Lizard, and Urodela | T |
| *Chironius multiventris* | 1 |  | T |
| *Chironius scurrulus* | 1 |  | T |
| *Dendrophidion dendrophis* | 1 |  | Arb |
| *Erythrolamprus pygmaeus* | 1 |  | T |
| *Erythrolamprus reginae* | 1 |  | T |
| *Erythrolamprus typhlus* | 1 |  | T |
| *Helicops angulatus* | 1 |  | Aq |
| *Imantodes cenchoa* | 1 |  | Arb |
| *Leptodeira annulata* | 1 |  | Arb |
| *Leptophis ahaetulla* | 1 |  | Arb |
| *Oxybelis aeneus* | 1 |  | Arb |
| *Oxyrhopus formosus* | 1 |  | T |
| *Philodryas argentea* | 1 |  | Arb |
| *Philodryas viridissima* | 1 |  | Arb |
| *Pseudoboa neuwiedii* | 1 |  | T |
| *Rhinobothryum lentiginosum* | 1 |  | T |
| *Siphlophis cervinus* | 1 |  | Arb |
| *Siphlophis compressus* | 1 |  | Arb |
| *Taeniophallus brevirostris* | 1 |  | SF |
| *Taeniophallus nicagus* | 1 |  | SF |
| *Xenodon rhabdocephalus* | 1 |  | T |
| *Xenopholis scalaris* | 1 |  | T |
| *Anilius scytale* | 2 | Amphisbaena, Fish, Gymnophiona, Myriapoda, Onychophore, and Snake | F |
| *Bothrops atrox* | 2 |  | SA |
| *Erythrolamprus aesculapii* | 2 |  | T |
| *Erythrolamprus breviceps* | 2 |  | T |
| *Helicops hagmanni* | 2 |  | Aq |
| *Micrurus averyi* | 2 |  | F |
| *Micrurus hemprichii* | 2 |  | F |
| *Micrurus lemniscatus* | 2 |  | SF |
| *Micrurus spixii* | 2 |  | SF |
| *Micrurus surinamensis* | 2 |  | Aq |
| *Tantilla melanocephala* | 2 |  | SF |
| *Boa constrictor* | 3 | Alligator, Bat, Bird eggs, Falconidae, Mammals (small, medium and big), Monkey, Passeriformes, Psittacidae, and Chelonian | SA |
| *Clelia clelia* | 3 |  | T |
| *Corallus caninus* | 3 |  | Arb |
| *Corallus hortulanus* | 3 |  | Arb |
| *Epicrates cenchria* | 3 |  | T |
| *Eunectes murinus* | 3 |  | Aq |
| *Lachesis muta* | 3 |  | T |
| *Oxybelis fulgidus* | 3 |  | Arb |
| *Oxyrhopus* aff *melanogenys* | 3 |  | T |
| *Phrynonax polylepis* | 3 |  | T |
| *Pseudoboa coronata* | 3 |  | T |
| *Spilotes pullatus* | 3 |  | Arb |
| *Spilotes sulphureus* | 3 |  | Arb |
| *Drepanoides anomalus* | 4 | Squamata eggs | T |
| *Drymoluber dichrous* | 4 |  | T |
| *Mastigodryas boddaerti* | 4 |  | T |
| *Dipsas indica* | 5 | Mollusk | Arb |
| *Dipsas pavonina* | 5 |  | Arb |

**Legend figures**

Figure A1: Histogram indicating the degree distribution (number of food interactions) between species of Amazonian snakes.

Figure A2: Network describing the interactions (lines) between species of Amazonian snakes (circles) and their food resources (triangle) categorized in a less specific way. 1. *Amerotyphlops reticulatus*; 2. *Anilius scytale*; 3. *Atractus alphonsehogei*; 4. *Atractus latifrons*; 5. *Atractus major*; 6. *Atractus poeppigi*; 7. *Atractus schach*; 8. *Atractus snethlageae*; 9. *Atractus torquatus*; 10. *Boa constrictor*; 11. *Bothrops atrox*; 12. *Chironius fuscus*; 13. *Chironius multiventris*; 14. Chironius scurrulus; 15. *Clelia clelia*; 16. *Corallus caninus*; 17. *Corallus hortulanus*; 18. *Dendrophidion dendrophis*; 19. *Dipsas indica*; 20. *Dipsas pavonina*; 21. Drepanoides anomalus; 22. *Drymoluber dichrous*; 23. *Epicrates cenchria*; 24. *Epictia tenella*; 25. *Erythrolamprus aesculapii*; 26. *Erythrolamprus breviceps*; 27. *Erythrolamprus pygmaeus*; 28. *Erythrolamprus reginae*; 29. *Erythrolamprus typhlus*; 30. *Eunectes murinus*; 31. *Helicops angulatus*; 32. *Helicops hagmanni*; 33. *Imantodes cenchoa*; 34. *Lachesis muta*; 35. *Leptodeira annulata*; 36. *Leptophis ahaetulla*; 37. *Mastigodryas boddaerti*; 38. *Micrurus averyi*; 39. *Micrurus hemprichii*; 40. *Micrurus lemniscatus*; 41. *Micrurus spixii*; 42. *Micrurus surinamensis*; 43. *Oxybelis aeneus*; 44. *Oxybelis fulgidus*; 45. *Oxyrhopus* aff. *melanogenys*; 46. *Oxyrhopus formosus*; 47. *Philodryas argentea*; 48. *Philodryas viridissima*; 49. *Phrynonax polylepis*; 50. *Pseudoboa coronate*; 51. *Pseudoboa neuwiedii*; 52. *Rhinobothryum lentiginosum*; 53. *Siphlophis cervinus*; 54. *Siphlophis* *compressus*; 55. *Spilotes pullatus*; 56. Spilotes sulphureus; 57. *Taeniophallus brevirostris*; 58. *Taeniophallus nicagus*; 59. *Tantilla melanocephala*; 60. *Typhlophis* *squamosus*; 61. *Xenodon* *rhabdocephalus*; 62. *Xenopholis* *scalaris*. X1. Mammals; X2. Rodents; X3. Bat; X4. Bird; X5. Fish; X6. Anuran; X7. Gymnophiona; X8. Anuran eggs; X9. Tadpoles; X10. Urodela; X11. Lizard; X12. Snake; X13. Chelonian; X14. Alligator; X15. Onychophoran; X16. Amphisbaena; X17. Elongated; X18. Bird eggs; X19. Squamata eggs; X20. Mollusk; X21. Earthworm; X22. Myriapoda; X23. Insect.

Figure A3: Network describing the interactions (lines) between species of Amazonian snakes (circles) and their food resources (triangle) categorized in a more specific way. 1. *Amerotyphlops reticulatus*; 2. *Anilius scytale*; 3. *Atractus alphonsehogei*; 4. *Atractus latifrons*; 5. *Atractus major*; 6. *Atractus poeppigi*; 7. *Atractus schach*; 8. *Atractus snethlageae*; 9. *Atractus torquatus*; 10. *Boa constrictor*; 11. *Bothrops atrox*; 12. *Chironius fuscus*; 13. *Chironius multiventris*; 14. Chironius scurrulus; 15. *Clelia clelia*; 16. *Corallus caninus*; 17. *Corallus hortulanus*; 18. *Dendrophidion dendrophis*; 19. *Dipsas indica*; 20. *Dipsas pavonina*; 21. Drepanoides anomalus; 22. *Drymoluber dichrous*; 23. *Epicrates cenchria*; 24. *Epictia tenella*; 25. *Erythrolamprus aesculapii*; 26. *Erythrolamprus breviceps*; 27. *Erythrolamprus pygmaeus*; 28. *Erythrolamprus reginae*; 29. *Erythrolamprus typhlus*; 30. *Eunectes murinus*; 31. *Helicops angulatus*; 32. *Helicops hagmanni*; 33. *Imantodes cenchoa*; 34. *Lachesis muta*; 35. *Leptodeira annulata*; 36. *Leptophis ahaetulla*; 37. *Mastigodryas boddaerti*; 38. *Micrurus averyi*; 39. *Micrurus hemprichii*; 40. *Micrurus lemniscatus*; 41. *Micrurus spixii*; 42. *Micrurus surinamensis*; 43. *Oxybelis aeneus*; 44. *Oxybelis fulgidus*; 45. *Oxyrhopus* aff. *melanogenys*; 46. *Oxyrhopus formosus*; 47. *Philodryas argentea*; 48. *Philodryas viridissima*; 49. *Phrynonax polylepis*; 50. *Pseudoboa coronate*; 51. *Pseudoboa neuwiedii*; 52. *Rhinobothryum lentiginosum*; 53. *Siphlophis cervinus*; 54. *Siphlophis* *compressus*; 55. *Spilotes pullatus*; 56. Spilotes sulphureus; 57. *Taeniophallus brevirostris*; 58. *Taeniophallus nicagus*; 59. *Tantilla melanocephala*; 60. *Typhlophis* *squamosus*; 61. *Xenodon* *rhabdocephalus*; 62. *Xenopholis* *scalaris*; X1. Bovidae; X2. Tayassuidae; X3. Suidae; X4. Cervidae; X5. Canidae; X6. Caviidae; X7. Atelidae; X8. Callitrichidae; X9. Cebidae; X10. Pitheciidae; X11. Dasypodidae; X12. Erethizontidae; X13. Mammals; X14. Marsupials; X15. Rodents; X16. Cricetidae; X17. Dasyproctidae; X18. Didelphidae; X19. Echimyidae; X20. Muridae; X21. Sciuridae; X22. Molossidae; X23. Phylostomatidae; X24. Vespertilionidae; X25. Falconidae; X26. Psittacidae; X27. Passeriformes; X28. Alcedinidae; X29. Bucconidae; X30. Caprimulgidae; X31. Columbidae; X32. Emberizidae; X33. Formicariidae; X34. Furnariidae; X35. Icteridae; X36. Phasianidae; X37. Pipridae; X38. Thamnophilidae; X39. Thraupidae; X40. Tyrannidae; X41. Fish; X42. Anostomidae; X43. Auchenipteridae; X44. Belonidae; X45. Callichthyidae; X46. Characidae; X47. Cichlidae; X48. Erythrinidae; X49. Gasteropelecidae; X50. Gymnotidae; X51. Heptapteridae; X52. Hypopomidae; X53. Lebiasinidae; X54. Pimelodidae; X55. Rivulidae; X56. Sciaenidae; X57. Synbranchidae; X58. Sternopygidae; X59. Anuran; X60. Allophrynidae; X61. Aromobatidae; X62. Bufonidae; X63. Craugastoridae; X64. Dendrobatidae; X65. Eleutherodactylidae; X66. Hylidae; X67. Leptodactylidae; X68. Microhylidae; X69. Anuran eggs; X70. Tadpole; X71. Ceciliidae; X72. Plethodontidae; X73. Lizard; X74. Alopoglossidae; X75. Corytophanidae; X76. Dactyloidae; X77. Gekkonidae; X78. Gymnophtalmidae; X79. Iguanidae; X80. Phyllodactylidae; X81. Polychrotidae; X82. Scincidae; X83. Sphaerodactylidae; X84. Teiidae; X85. Tropiduridae; X86. Snake; X87. Aniliidae; X88. Boidae; X89. Colubridae; X90. Dipsadidae; X91. Elapidae; X92. Leptotyphlopidae; X93. Typhlopidae; X94. Amphisbaena; X95. Chelonian; X96. Alligatoridae; X97. Bird eggs; X98. Squamata eggs; X99. Onychophoran; X100. Snail; X101. Slug; X102. Earthworms; X103. Myriapoda; X104. Acari; X105. Ant eggs and pupae; X106. Insect.

Figure A4: The number of categories of food resources consumed by different snakes species of the Dipsadidae family is positively associated with the snake average body mass (slope = 0.75, R^2^ = 0.17, p = 0.02) in a network of interactions between Amazonian snakes and their resources. Each point represents a species. To avoid overlap, some points have been slightly offset from their original position on the x-axis.

Figure A5: The number of categories of food resources consumed by different snakes species of the Colubridae family is positively associated with the snake average body mass (slope = 1.26, R^2^ = 0.25, p = 0.04) in a network of interactions between Amazonian snakes and their resources. Each point represents a species. To avoid overlap, some points have been slightly offset from their original position on the x-axis.

Figure A6: As contained in Figure 1 of the main text of the manuscript, this figure describes the network of interactions (lines) between the species of snakes in the Amazon (circles) and their food resources (triangle), including the names of the snake species and the names of the resource categories. 1. *Amerotyphlops reticulatus*; 2. *Anilius scytale*; 3. *Atractus alphonsehogei*; 4. *Atractus latifrons*; 5. *Atractus major*; 6. *Atractus poeppigi*; 7. *Atractus schach*; 8. *Atractus snethlageae*; 9. *Atractus torquatus*; 10. *Boa constrictor*; 11. *Bothrops atrox*; 12. *Chironius fuscus*; 13. *Chironius multiventris*; 14. Chironius scurrulus; 15. *Clelia clelia*; 16. *Corallus caninus*; 17. *Corallus hortulanus*; 18. *Dendrophidion dendrophis*; 19. *Dipsas indica*; 20. *Dipsas pavonina*; 21. Drepanoides anomalus; 22. *Drymoluber dichrous*; 23. *Epicrates cenchria*; 24. *Epictia tenella*; 25. *Erythrolamprus aesculapii*; 26. *Erythrolamprus breviceps*; 27. *Erythrolamprus pygmaeus*; 28. *Erythrolamprus reginae*; 29. *Erythrolamprus typhlus*; 30. *Eunectes murinus*; 31. *Helicops angulatus*; 32. *Helicops hagmanni*; 33. *Imantodes cenchoa*; 34. *Lachesis muta*; 35. *Leptodeira annulata*; 36. *Leptophis ahaetulla*; 37. *Mastigodryas boddaerti*; 38. *Micrurus averyi*; 39. *Micrurus hemprichii*; 40. *Micrurus lemniscatus*; 41. *Micrurus spixii*; 42. *Micrurus surinamensis*; 43. *Oxybelis aeneus*; 44. *Oxybelis fulgidus*; 45. *Oxyrhopus* aff. *melanogenys*; 46. *Oxyrhopus formosus*; 47. *Philodryas argentea*; 48. *Philodryas viridissima*; 49. *Phrynonax polylepis*; 50. *Pseudoboa coronate*; 51. *Pseudoboa neuwiedii*; 52. *Rhinobothryum lentiginosum*; 53. *Siphlophis cervinus*; 54. *Siphlophis* *compressus*; 55. *Spilotes pullatus*; 56. Spilotes sulphureus; 57. *Taeniophallus brevirostris*; 58. *Taeniophallus nicagus*; 59. *Tantilla melanocephala*; 60. *Typhlophis* *squamosus*; 61. *Xenodon* *rhabdocephalus*; 62. *Xenopholis* *scalaris*. X1. Big mammals; X2. Monkey; X3. Medium mammals; X4. Small mammals; X5. Bat; X6. Falconidae; X7. Psittacidae; X8. Passeriformes; X9. Fish; X10. Anuran; X11. Anuran eggs and tadpole; X12. Gymnophiona; X13. Urodela; X14. Lizard; X15. Snake; X16. Amphisbaena; X17. Chelonian; X18. Alligator; X19. Bird eggs; X19. Squamata eggs; X20. Onychophore; X21. Mollusk; X22. Earthworm; X23. Myriapoda; X25. Acari; X26. Insect.

Figure A7: Ordination graph by correspondence analysis (CA), showing snake species (blue color labels) and food resources categories (red color labels) conventionally plotted on the first two ordination axes (in parenthesis the total variance explained by the axis). To avoid overlap, some labels have been slightly offset from their original position.

**Complementary references of the diet of Amazonian snakes referring to the databank in Appendix 2 Table A1.**

Albarelli, L. P. P. and Santos-Costa, M. C. 2010. Feeding ecology of *Liophis reginae* *semilineatus* (Serpentes: Colubridae: Xenodontinae) in Eastern Amazon, Brazil. - Zoologia 27(1): 87–91.

Bernarde, P. S. and Abe, A. S. 2010. Hábitos alimentares de serpentes em Espigão do Oeste, Rondônia, Brasil. - Biota Neotrop. 10(1): 167-173.

Bernarde, P. S. et al. 2017. Serpentes do Alto Juruá, Acre - Amazônia brasileira. - Rio Branco - Acre: Edufac.

Capurucho, J. M. G. and Costa, H. C. 2012. *Oxybelis fulgidus*. Diet. Natural History Notes. – Herpetol. Rev. 43(3): 495.

Crnobrna, B. et al. 2016. *Drepanoides anomalus*. Diet/Ophiophagy. Natural History Notes. - Herpetological Review 47(3): 478.

Cunha, F. A. G. and Vogt, R. C. 2016. Anilius scytale. Diet. Natural history notes. - Herpetol. Rev. 47(1), 141.

Eversole, C. B. et al. 2018. *Leptophis ahaetulla*. Diet. Natural History Notes. - Herpetol. Rev. 49(2): 345.

Gama, C. E. S. et al. 2018. *Oxybelis fulgidus*. Diet. Natural History Notes. – Herpetol. Rev. 49(4): 759.

Hernández-Ruz, E. J. 2019. *Oxybelis fulgidus*. Diet. Natural History Notes. – Herpetol. Rev. 50(1): 166.

Jorge, R. F. and Simões, P. I. 2018. *Philodryas viridissima*. Diet. Natural History Notes. – Herpetol. Rev. 49(4): 762.

Kaefer, I. L. and Montanarin A. 2011. *Helicops angulatus*. Diet. Natural History Notes. - Herpetol. Rev. 42(2): 291.

Leite, G. A. and Dorado-Rodrigues, T. F. 2017. *Epicrates cenchria*. Diet. Natural History Notes. – Herpetol. Rev. 48(2): 450.

Macedo-Bernarde, L. C. 2006. *Eunectes murinus* (Linnaeus) (Serpentes, Boidae), preying activity. - Pan-Am. J. aquat. sci. 1(2): II.

Maschio, G. F. et al. 2010. Food habits of *Anilius scytale* (Serpentes: Aniliidae) in the Brazilian Amazonia. - Zoologia 27(2): 184–190.

Mendes-Júnior, R. N. G. et al. 2013. *Helicops angulatus*. Diet/Scavenging. Natural History Notes. - Herpetol. Rev. 44(2): 330.

Pena, A. S. et al. 2017. *Oxybelis fulgidus*. Diet. Natural History Notes. - Herpetol. Rev. 48(1): 217.

Pinto, R. R. et al. 2011. *Micrurus surinamensis*. Ophiophagy. – Herpetol. Rev. 42(3): 441.

Pizzato, L. P. et al. 2009. Food habits of Brazilian boid snakes: overview and new data, with special reference to *Corallus hortulanus*. - Amphibia-Reptilia 30: 533-544.

Prado, P. C. et al. 2016. *Erythrolamprus typhlus*. Diet. Natural History Notes. – Herpetol. Rev. 47(3): 478.

Prudente, A. L. C. et al. 2007. Morphology, Reproductive Biology and Diet of *Dendrophidion dendrophis* (Schlegel, 1837) (Serpentes, Colubridae) in Brazilian Amazon. – S. Am. J. Herpetol. 2(1): 53-58.

Ribeiro-Junior, M. A. et al. 2016. Predation of a squirrel monkey (*Saimiri sciureus*) by an Amazon tree boa (*Corallus hortulanus*): even small boids may be a potential threat to small-bodied platyrrhines. - Primates 57: 317–322.

Rivas, J. A. 2004. *Eunectes murinus* (green anaconda): Subduing behavior. - Herpetol. Rev. 35(1): 66-67.

Rivas, J. A. and Owens, R. Y. 2000. *Eunectes murinus* (green anaconda): Cannibalism. - Herpetol. Rev. 31(1): 45-46.

Rivas, J. A. et al. 1999. Natural History Notes. *Eunectes murinus* (Green Anaconda). *Caiman* Predation. - Herpetol. Rev. 30: 101.

Silva, M. V. et al. 2010. Riqueza e dieta de serpentes do Estado do Acre, Brasil. - Rev. Bras. Zoociências 12(2): 165-176.

Silva, G. D. and Costa-Campos, C. E. 2016. *Erythrolamprus aesculapii* Diet. Natural History Notes. - Herpetol. Rev. 47(4): 681.

Silva-Ferreira, D. S. et al. 2017. *Chironius fuscus*. Diet. Natural History Notes. - Herpetol. Rev. 48(1): 206.

Sousa, K. R. M. et al. 2014. Reproduction and diet of *Imantodes cenchoa* (Dipsadidae: Dipsadinae) from the Brazilian Amazon. - Zoologia 31(1): 8–19.

Tavares-Pinheiro, R. et al. 2019. *Helicops angulatus*. Diet. Natural History Notes. - Herpetol. Rev. 50(1): 157.

Teixeira, C. C. 2017. Diet Composition and Foraging Habitat Use by Three Species of Water Snakes, *Helicops* Wagler, 1830, (Serpentes: Dipsadidae) in Eastern Brazilian Amazonia. - J. Herpetol. 51(2): 215-222.

Viana, P. F. et al. 2014. *Oxybelis fulgidus*. Diet. Natural History Notes. - Herpetol. Rev. 45(3): 519.

Vieira, B. P. and Gomes, F. B. R. 2016. *Leptophis ahaetulla*. Diet. Natural History Notes. - Herpetol. Rev. 47(1): 147.
